# Ability of detecting and willingness to share fake news

**DOI:** 10.1038/s41598-023-34402-6

**Published:** 2023-05-05

**Authors:** K. Peren Arin, Deni Mazrekaj, Marcel Thum

**Affiliations:** 1 Zayed University, Abu Dhabi, United Arab Emirates; 2 Centre for Applied Macroeconomic Analysis, Canberra, Australia; 3 Utrecht University, Utrecht, The Netherlands; 4 University of Oxford, Oxford, UK; 5 KU Leuven, Leuven, Belgium; 6 TU Dresden, Dresden, Germany; 7 ifo Dresden, Dresden, Germany; 8 CESifo, Munich, Germany

**Keywords:** Health occupations, Health care

## Abstract

By conducting large-scale surveys in Germany and the United Kingdom, we investigate the individual-level determinants of the ability to detect fake news and the inclination to share it. We distinguish between deliberate and accidental sharing of fake news. We document that accidental sharing is much more common than deliberate sharing. Furthermore, our results indicate that older, male, high-income, and politically left-leaning respondents better detect fake news. We also find that accidental sharing decreases with age and is more prevalent among right-leaning respondents. Deliberate sharing of fake news is more prevalent among younger respondents in the United Kingdom. Finally, our results imply that respondents have a good assessment of their ability to detect fake news: those we identified as accidental sharers were also more likely to have admitted to having shared fake news.

## Introduction

Fake news is pernicious as it spreads misleading and false information. Lazer et al.^1^ distinguish between misinformation (false or misleading information) and disinformation (false information that is purposely spread to deceive people). In both information contexts, fake news is likely to promote misperceptions. Recent studies show that the information overload during the COVID-19 pandemic increased the likelihood of fake news sharing by increasing consumers’ psychological strain^2,3^. By altering people’s perceptions and attitudes, fake news can shape public debates about critical policy issues such as vaccinations or immigration. For instance, Allcott and Gentzkow^4^ suggest that in the 2016 presidential election in the United States, fake news had a considerable impact and could even have been crucial in determining the election’s outcome. Brady et al.^5^ contend that moral-emotional language in political messages substantially increases their diffusion within (and less so between) ideological group boundaries.

In this paper, we conduct large-scale surveys in Germany and the United Kingdom to shed light on the individual-level determinants of the ability to detect fake news and the inclination to spread it, either deliberately or accidentally. The spread of fake news is becoming a public and global concern^1^. Fake news spreads “significantly farther, faster, deeper, and more broadly than the truth”^6^. According to Barthel, Mitchell, and Holcomb^7^, about two-in-three US adults state that fake news causes a great deal of confusion about contemporary issues and events. This perceived confusion is widely shared across incomes, education levels, partisan affiliations, and other demographic characteristics.

Academic literature lacks agreement on which individual factors contribute to the difficulty in detecting fake news and the tendency to spread it. Regarding detection, Allcott and Gentzkow^4^ found that partisan individuals have more difficulty identifying fake news that confirms their political stances. After the 2016 US presidential election, partisan individuals were more likely to believe headlines that favored their preferred candidate. This ideological bias was substantially more robust for those who relied on ideology-based social media networks. Bail et al.^8^ conducted a field experiment that offered a large group of Democrats and Republicans financial compensation to follow bots that retweeted messages by elected officials and opinion leaders with opposing political views. Surprisingly, Republican participants expressed substantially more conservative views after following a liberal Twitter bot. Pennycook and Rand^9^ concluded that it is primarily analytical thinking that matters for assessing of news headlines independent of whether a headline matches one’s political ideology. In the United Kingdom, Preston et al.^10^ found that higher levels of educational attainment and emotional intelligence go along with a better ability to detect fake news. However, there is still a lack of systematic evidence on the effect of age and other individual-level characteristics such as education, gender, or income on the ability to detect fake news. Such evidence may help target particularly vulnerable groups with appropriate policy measures such as fake news alerts that remind viewers of potentially misleading or false information.

Not all individuals who encounter fake news are also spreading it. Grinberg et al.^11^ examined the sharing of fake news in the US 2016 election and found that engagement with fake news sources was highly concentrated among specific sub-populations. Individuals most likely to engage with fake news sources were conservative-leaning, older, and those who were highly engaged in political news. Hopp, Ferucci and Vargo^12^ identified people with politically extreme views and distrust in mainstream media as the main disseminators of fake news. In a similar vein, Guess, Nagler, and Tucker^13^ found that conservative people and those older than 65 are more likely to share pro-Trump fake news. Older people are also over-represented among super-spreaders of fake news^11^. By contrast, Buchanan^14^ found that younger, male, and less educated individuals were more likely to spread disinformation they encountered on social media in the UK. In general, men share more unverified information^15^. Compared to the ability to detect fake news, more studies on sharing fake news have investigated individual factors. However, most of these studies focused on the United States. This immediately raises the question of whether findings are similar or different in politically less polarized countries and where a significant part of political and societal information comes from highly reputed nationwide public broadcasting (such as the BBC in the UK or ARD and ZDF in Germany).

Moreover, most previous studies did not differentiate whether fake news is shared deliberately or accidentally. The distinction is crucial when it comes to policy conclusions. If fake news is spread mostly accidentally, then the policy can focus on citizens’ ability to identify fake news. If fake news is mostly shared deliberately, improving identification will not help; a broader approach to overcoming the societal divide may be needed.

The contribution of the paper is twofold. First, our survey allows for comparing fake news detection and sharing across two major European countries: Germany and the United Kingdom. In contrast, the majority of the literature so far has focused on the United States. In their systematic literature review of 45 experimental studies that investigate the determinants of identifying fake news, Bryanov and Vziatysheva^16^ write: “The sample is heavily skewed towards the US” and “The comparative aspect of fake news perceptions, too, is conspicuously understudied.” Among the 45 articles reviewed, there is only one comparative study (US–India), and just three studies focus on a country other than the US. Even when taking into account the most recent publications and considering the broader literature using purely self-reported behavior regarding fake news, the focus is mainly on the US; exceptions are, e.g., Apuke and Omar^3^ for Nigeria, Islam et al.^17^ and Laato et al.^15^ for Bangladesh, Buchanan^14^ and Preston et al.^10^ for the UK.

For the comparative analysis, we conducted large-scale surveys in Germany (1223 respondents) and the United Kingdom (1156 respondents); see Table 1. The comparison between Germany and the UK is particularly interesting as the UK has experienced a wave of populist policies in the wake of Brexit—mirrored in the media landscape. In contrast, these populist debates have played a minor role in Germany. Hence, there may be a broader acceptance of fact-ignoring arguments in the UK. It is also useful to study these countries because of the extensive debate in Germany about limiting the spread of misinformation and hate via social media. Within four years, three bills passed in the German parliament that aimed to combat hate crimes more effectively, punishing false news and criminal content on social network platforms. For instance, social networks must report on their dealing with hate crimes and maintain an effective complaint management system. Even though the law did not address fake news in general but only the spread of hate via fake news, the controversial public debate around these government regulations^18^ may have sharpened the people’s senses when encountering fake news in general. Such a public debate was absent in the UK. Consequently, we hypothesize that there is less detection and more fake news sharing in the UK compared to Germany. This should be mostly a level effect, as it is unclear why particular sociodemographic groups would be affected differently by the public debates in the two countries.

**Table 1:** Descriptive statistics (N = 2379).

	Germany				United Kingdom			
	Population	Mean/Prop.	Min.	Max.	Population	Mean/Prop.	Min.	Max.
Female	.496	.500	0	1	.503	.446	0	1
Age	45.2	48.6	19	70	43.8	52.4	20	70
High educated	.280	.398	0	1	.433	.452	0	1
Married/cohabiting	.611	.428	0	1	.631	.389	0	1
Low income	.244	.177	0	1	.270	.257	0	1
Middle income	.594	.610	0	1	.430	.537	0	1
High income	.163	.206	0	1	.300	.206	0	1
Employed	.754	.801	0	1	.753	.702	0	1
Unemployed	.023	.015	0	1	.024	.018	0	1
Out of labor force	.222	.184	0	1	.223	.279	0	1
Left		.214	0	1	.221	.189	0	1
Center		.645	0	1	.634	.593	0	1
Right		.141	0	1	.145	.217	0	1
Detecting fake news		23.662	12	38		22.835	12	37
Sharing fake news		.635	0	10		1.065	0	10
Deliberate sharing		.195	0	5		.231	0	5
Accidental sharing		.365	0	5		.623	0	5
Admits deliberate sharing		.035	0	1		.077	0	1
Admits accidental sharing		.061	0	1		.090	0	1

This table shows summary statistics from our sample alongside representative statistics of population in each country. Data for gender, age, employed, household type and unemployed come from Eurostat. Eurostat is the statistical office of the European Union: https://ec.europa.eu/eurostat/. “Married/Coh.” captures the share of the adult population living as a couple; the data for the entire population is taken from the Labor Force Statistics ($LFST \_ HHNHTYCH$, number of private households by household composition). The education data also comes from the Labor Force survey ($LFSA \_ PGAED$, population by sex, age and educational attainment level) and refers to the population aged 20–64. For income data the sources are: (1) For Germany: National Statistics Institute (https://www.destatis.de/DE/Home/_inhalt.html). Income levels (monthly net household income) are: less than 1500€; 1500€–2999€; 3000€ or more; (2) For the United Kingdom: National Statistics Institute (https://www.gov.uk/search/research-and-statistics). Income levels (gross weekly household income) are: less than £400; £400–£1000; £1000 or more. Employment data is taken from the labor force survey (population by sex, age, citizenship and labor status, $LFSQ\_PGANWS$). The employed category also includes self-employed.

Our second contribution is that we simultaneously study detecting as well as accidental and deliberate sharing of fake news in an experimental setting. Deliberate sharing means that respondents know that the news they are willing to share is unlikely to be true; accidental sharing occurs if respondents share fake news, which they erroneously believe to be true. To distinguish between accidental and deliberate sharing, we must elicit whether respondents can correctly identify fake news items. Several papers focused on either the detection^19–23^ or on the sharing of fake news^3,11,12,15,17,24^, but not both. Further, Buchanan^14^ distinguishes between accidental and deliberate sharing of fake news but does so with a retrospective survey question only. Accidental and deliberate sharing is not measured through actual sharing behavior or through the intention to share an actual news item. Instead, respondents are asked in retrospect whether they have ever shared fake news accidentally or deliberately. We gave the respondents ten viral news headlines from the internet, five being “fake news” and the other five being true news stories. We selected headlines that avoided any national bias for the UK or Germany. None of the headlines deal with domestic affairs in Germany or the UK as this would facilitate the validation for respondents from the respective country. We also avoided headlines associated with national policy stances to avoid any partisan bias. We pretested the headlines among Ph.D. students to ensure sufficient variation in response.

The headlines were presented as a text and not combined with any visual material to avoid deception, e.g., falsely suggesting the appearance in specific (social) media outlets. We asked respondents to assess these headlines and created an index that quantifies their ability to detect fake news (for details, see “Methods” Section). We also asked respondents how likely they would spread each of the ten news headlines. By comparing the identification of fake news items with the intention to share them, we can distinguish accidental and deliberate sharing. We hypothesize that most sharing is due to a lack of detection, i.e., accidental sharing dominates. Recent research has shown that most people are overconfident in their own ability to spot fake news^25^. The overconfidence in one’s own ability to distinguish true and false information may contribute to spreading fake news^26^. Differences in overconfidence may explain why some groups fall more easily for fake news. As overconfidence is generally higher among men^27^, particularly in tasks with low-performance feedback^28^ such as news sharing, accidental sharing should be higher among male respondents. We also expect more accidental sharing among the younger individuals^29^.

Finally, we asked the retrospective questions of whether the respondents had shared fake news stories accidentally or deliberately in the past; this allowed us to check whether respondents had a good assessment of their sharing behavior. We compare the responses to the retrospective questions with the actual behavior in the experiment with the headlines. Due to the general overconfidence, we expect a low correlation between actual and perceived accidental sharing, i.e., low awareness of being a distributor of fake news. With respect to deliberate sharing, we hypothesize that respondents are frank about their behavior. Those who do not admit their deliberate sharing for reasons of social desirability will also mask this behavior in the experimental setting.

## Results

### Individual determinants of fake news detection and sharing

Table 2 presents pairwise correlations between the variables, while Table 3 displays regression results for the individual-level determinants of two fake news indicators: (a) how good respondents are in separating fake news from accurate ones (Column 1 for Germany and Column 2 for the United Kingdom); and (b) how likely they are to spread fake news stories (Columns 3 and 4). We will focus on the regression results because we can measure the relationship between variables net of the other variables and we can also make the interpretation more explicit. The outcome in the first two columns is a scale constructed from the number of headlines correctly identified by a respondent. A higher value indicates better detection (see “Methods” Section). In the first two columns, we explain the respondents’ ability to identify fake news headlines with various individual-level factors including the respondent’s political orientation. The outcome is replaced in the last two columns by a scale constructed from the number of fake statements a respondent is willing to spread. Hence, we explain the willingness to spread misinformation (deliberately or accidentally) with individual-level variables.

**Table 2:** Pairwise correlations.

Variables	(1)	(2)	(3)	(4)	(5)	(6)	(7)	(8)	(9)	(10)	(11)	(12)	(13)	(14)
(1) Country	1.000													
(2) Gender	− 0.054	1.000												
	(0.008)													
(3) Age	0.153	− 0.105	1.000											
	($< 0.001$)	($< 0.001$)												
(4) Education	0.055	0.027	− 0.137	1.000										
	(0.007)	(0.181)	($< 0.001$)											
(5) Marital status	− 0.039	0.037	− 0.050	− 0.068	1.000									
	(0.057)	(0.071)	(0.014)	(0.001)										
(6) Household income	− 0.062	− 0.057	− 0.093	0.218	− 0.381	1.000								
	(0.003)	(0.005)	($< 0.001$)	($< 0.001$)	($< 0.001$)									
(7) Labour market position	0.115	0.062	0.332	− 0.141	0.069	− 0.255	1.000							
	($< 0.001$)	(0.003)	($< 0.001$)	($< 0.001$)	(0.001)	($< 0.001$)								
(8) Political orientation	0.082	− 0.044	− 0.005	− 0.045	− 0.084	0.081	− 0.070	1.000						
	($< 0.001$)	(0.030)	(0.789)	(0.029)	($< 0.001$)	(0.000)	(0.001)							
(9) Detecting fake news	− 0.119	− 0.125	0.099	0.043	− 0.008	0.088	0.003	− 0.074	1.000					
	($< 0.001$)	($< 0.001$)	($< 0.001$)	(0.037)	(0.692)	($< 0.001$)	(0.902)	($< 0.001$)						
(10) Sharing fake news	0.114	− 0.058	− 0.134	− 0.001	− 0.031	0.016	− 0.074	0.126	− 0.270	1.000				
	($< 0.001$)	(0.004)	($< 0.001$)	(0.964)	(0.134)	(0.437)	($< 0.001$)	($< 0.001$)	($< 0.001$)					
(11) Deliberate sharing	0.026	− 0.047	− 0.072	− 0.019	− 0.009	0.037	− 0.022	0.024	− 0.084	0.542	1.000			
	(0.209)	(0.022)	(0.000)	(0.365)	(0.656)	(0.074)	(0.286)	(0.251)	($< 0.001$)	($< 0.001$)				
(12) Accidental sharing	0.104	− 0.042	− 0.115	− 0.009	− 0.021	− 0.010	− 0.071	0.119	− 0.296	0.866	0.150	1.000		
	(0.000)	(0.041)	($< 0.001$)	(0.675)	(0.306)	(0.632)	(0.001)	($< 0.001$)	($< 0.001$)	($< 0.001$)	($< 0.001$)			
(13) Admits accidental sharing	0.054	− 0.067	− 0.102	0.055	− 0.020	0.014	− 0.063	0.089	− 0.091	0.346	0.124	0.305	1.000	
	(0.008)	(0.001)	($< 0.001$)	(0.007)	(0.326)	(0.493)	(0.002)	($< 0.001$)	($< 0.001$)	($< 0.001$)	($< 0.001$)	($< 0.001$)		
(14) Admits deliberate sharing	0.091	− 0.039	− 0.126	0.070	− 0.056	0.046	− 0.063	0.093	− 0.118	0.394	0.172	0.330	0.613	1.000
	($< 0.001$)	(0.058)	($< 0.001$)	(0.001)	(0.006)	(0.025)	(0.002)	($< 0.001$)	($< 0.001$)	($< 0.001$)	($< 0.001$)	($< 0.001$)	($< 0.001$)	

*p* values are between parentheses.

**Table 3:** Detecting and sharing fake news by country.

	Detecting fake news		Sharing fake news	
	(1)	(2)	(3)	(4)
	Germany	UK	Germany	UK
Female (ref:male)	− 1.187***	− 0.451*	− 0.051	− 0.408**
	(0.199)	(0.202)	(0.090)	(0.125)
Age	0.044***	0.021*	− 0.011**	− 0.040***
	(0.008)	(0.009)	(0.004)	(0.006)
High educated (ref:low edu)	0.544*	0.028	− 0.157+	− 0.037
	(0.212)	(0.205)	(0.087)	(0.128)
Separated/Single (ref:married/coh)	0.187	0.207	− 0.038	− 0.204
	(0.220)	(0.220)	(0.097)	(0.130)
Middle income (ref:low)	0.131	0.499*	− 0.007	− 0.047
	(0.287)	(0.237)	(0.124)	(0.142)
High income (ref:low)	0.880*	0.954**	− 0.132	− 0.062
	(0.386)	(0.326)	(0.157)	(0.195)
Unemployed (ref:employed)	− 1.131	0.062	0.599	− 0.441
	(0.840)	(0.748)	(0.635)	(0.375)
Out of labor force (ref:employed)	− 0.024	0.197	− 0.112	− 0.116
	(0.271)	(0.237)	(0.099)	(0.132)
Center (ref:left)	− 0.602*	− 0.889***	0.006	0.346*
	(0.252)	(0.261)	(0.099)	(0.143)
Right (ref:left)	− 0.372	− 1.027**	0.320+	1.027***
	(0.352)	(0.314)	(0.169)	(0.199)
Constant	22.031***	22.050***	1.273***	3.111***
	(0.609)	(0.601)	(0.255)	(0.414)
N	1223	1156	1223	1156
Adj. R-sq	0.070	0.021	0.015	0.074

All estimates are from linear models estimated by Ordinary Least Squares. HC1 robust standard errors are in parentheses. + *p* $<0.10$, * *p* $<0.05$, ** *p* $<0.01$, *** *p* $<0.001$ (two-tailed tests).

Our results show that high-income and older respondents are better able to detect fake news in both countries; female respondents and centrists are worse at it (Columns 1 and 2). These four demographic characteristics are statistically significant in both countries. The only individual-level factors that show different impacts in the two countries are education and right-wing orientation: those with higher education in Germany have a better ability to detect fake news, while in the United Kingdom, the ability does not depend on education levels. Right-wing respondents in the UK are worse at detecting fake news compared to left-wing respondents; no such effect is detectable in Germany. When it comes to sharing fake news (Columns 3 and 4), the young and the politically right-leaning respondents are more likely to distribute fake news in both countries. In the United Kingdom, female respondents are less and centrists are more likely to distribute fake news; in Germany, these individual-level variables are insignificant.

We can also investigate the country differences in more detail. Supplement Table 2 repeats the regressions for detecting and sharing fake news, but here the data for the two countries are pooled, and a country dummy is added. In line with our hypothesis, UK respondents detect fake news less accurately and share it more frequently. Supplement Fig. 1 in the Online Appendix illustrates the differences between the two countries regarding the ability to detect fake news in a box plot. We have also tested whether there are any statistically significant differences between the coefficients of individual-level determinants in the two countries for the regressions without the country dummy presented in Table 3. For fake news detection, the Wald test of equality of coefficients suggests that only gender coefficients are significantly different between the two countries at the 5% level (*p* = 0.009). Thus, although women detect fake news less frequently than men in both countries, the difference between women and men in detecting fake news is significantly larger in Germany than in the UK. For fake news sharing, gender (*p* = 0.020), age (0.000), and both political orientation coefficients (*p* = 0.050 and *p* = 0.006) are significantly different between the two countries.

### Who shares fake news deliberately or accidentally?

We next differentiate between deliberate and accidental fake news sharing. We have constructed two scales based on the five fake news statements. The first scale is deliberate sharing meaning a respondent recognizes fake news but wants to share it anyway. The higher the number, the more deliberate sharing there is; the maximum is five meaning that a respondent identified five headlines as fake and wanted to share all of them. The second scale refers to accidental sharing; it measures the number of fake news statements that were not recognized as fake, and the respondent wanted to share them.

**Figure 1:**
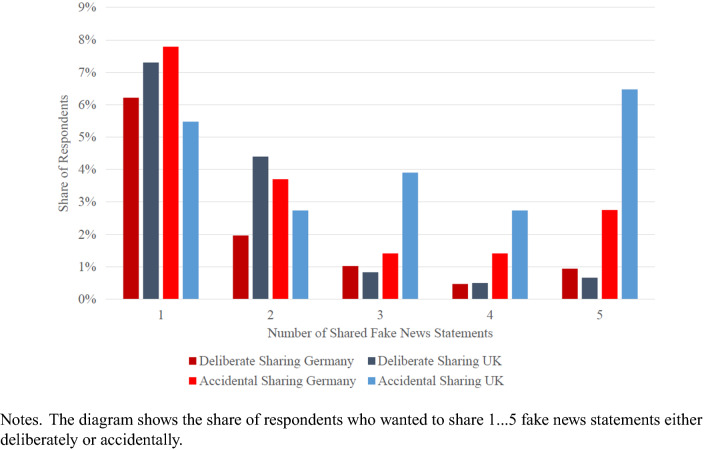
Share of Respondents Sharing Fake News Statements.

Figure 1 displays the share of respondents who would like to share at least one fake-news statement. The percentages are presented separately for deliberate and accidental sharing and for the two countries. There is more accidental than deliberate sharing. Around 12 percent of respondents want to share at least one news item that they have correctly identified as fake news. In Supplement Table 1, we observe that these respondents are disproportionately from the UK, male, and right on the political spectrum compared to the respondents who did not share any fake news. More than 19 percent (17 percent in Germany, 21 percent in the UK) want to share accidentally, i.e., at least one of the news items they want to share is not correctly identified as fake news. A striking difference emerges in the percentage of respondents who accidentally share a large number of fake news headlines. In Germany, only 6 percent accidentally share three or more fake news headlines, whereas the percentage is 13 percent in the United Kingdom.

Table 4 shows the individual-level determinants for deliberate (columns 1 and 2) and accidental sharing (columns 3 and 4) in Germany and the United Kingdom. There is no clear pattern for deliberate sharing. There is only a statistically significant higher tendency for deliberate sharing among younger people in the United Kingdom. It should be stressed that the overall number of people who correctly identify fake news and still want to distribute it is relatively small. In contrast, the numbers for accidental sharing are much higher. Here we find consistently across the two countries that right-wing respondents share more frequently and accidental sharing decreases with age. In the United Kingdom, women share less accidentally than men; here, centrist respondents have a higher tendency for accidental fake news than the reference group of left-wing respondents.

**Table 4:** Deliberate and accidental sharing of fake news by country.

	Deliberate sharing		Accidental sharing	
	(1)	(2)	(3)	(4)
	Germany	UK	Germany	UK
Female (ref:male)	− 0.066	− 0.076+	0.002	− 0.225**
	(0.044)	(0.041)	(0.059)	(0.081)
Age	− 0.002	− 0.009***	− 0.007**	− 0.021***
	(0.002)	(0.002)	(0.002)	(0.004)
High educated (ref:low edu)	− 0.030	− 0.078+	− 0.121*	− 0.006
	(0.042)	(0.041)	(0.059)	(0.082)
Separated/Single (ref:married/coh)	0.030	− 0.036	− 0.067	− 0.078
	(0.045)	(0.042)	(0.067)	(0.087)
Middle income (ref:low)	0.004	0.019	− 0.031	− 0.071
	(0.059)	(0.048)	(0.085)	(0.098)
High income (ref:low)	0.037	0.124+	− 0.164	− 0.164
	(0.076)	(0.069)	(0.106)	(0.128)
Unemployed (ref:employed)	− 0.003	0.153	0.231	− 0.357+
	(0.128)	(0.179)	(0.307)	(0.215)
Out of labor force (ref:employed)	− 0.003	0.036	− 0.094	− 0.119
	(0.053)	(0.054)	(0.065)	(0.091)
Center (ref:left)	− 0.057	0.033	0.094	0.227*
	(0.054)	(0.049)	(0.060)	(0.094)
Right (ref:left)	− 0.046	0.113+	0.239*	0.596***
	(0.065)	(0.064)	(0.101)	(0.125)
Constant	0.362**	0.679***	0.770***	1.702***
	(0.126)	(0.123)	(0.158)	(0.273)
N	1223	1156	1223	1156
Adj. R− sq	0.000	0.023	0.016	0.050

Outcome variables are scales ranging from 0 to 5 with a higher value indicating more sharing of fake news. All estimates are from linear models estimated by Ordinary Least Squares. HC1 robust standard errors are in parentheses. + *p* $<0.10$, * *p* $<0.05$, ** *p* $<0.01$, *** *p* $<0.001$ (two-tailed tests).

Accidental and deliberate sharing may also be driven by general sharing behavior. Those who never share any news are also not at risk of sharing fake news. To assess the extent of sharing in general, we estimate regressions where we explain the general sharing behavior with individual-level characteristics. We use the number of true headlines that a respondent wants to share as a proxy for general sharing behavior (see Supplement Table 3). Sharing activity decreases with age; right-leaning and male respondents tend to share more, especially in the UK. Hence, some of the significant individual-level drivers of accidental sharing, which we identified earlier, may indeed be partially driven by general sharing behavior.

### How do people assess their sharing behavior?

The previous sections focused on objective measures of sharing fake news as they were based on the number of fake news items people were willing to forward deliberately or accidentally. This section analyzes the respondents’ self-perception of how they deal with fake news. For this purpose, we asked whether people have shared a political news story online that they later found out to be made up (accidental sharing) or that they thought at the time to be made up (deliberate sharing). Again we are interested in the individual-level characteristics that explain the accidental and deliberate sharing of fake news. Therefore, in addition to the standard individual-level characteristics, we include our objective measures of deliberate and accidental fake news sharing as an explanatory variable. This allows us to answer whether those who shared more fake news are also aware of their behavior. And it allows us to assess whether the answers to the retrospective question of past sharing behavior—common in the literature—match with actual sharing behavior observed in our experiment.

The results are displayed in Table 5. We explain the respondents’ perception of their sharing behavior with the actual sharing behavior, which we elicited from the fake news statements in the survey, and the various individual-level characteristics. The results for the deliberate sharing of fake news are in Column 1 for Germany and in Column 2 for the United Kingdom; the corresponding results for the accidental sharing of fake news are in Columns 3 and 4. The key insight is that those who share more fake news also admit to doing so. This is true for accidental and deliberate sharing. Hence, people are quite aware of their weakness in spotting fake headlines (accidental sharing). For deliberate sharing, the actual behavior matches very well with the answers to the retrospective question. Our results also imply that for a given level of deliberate sharing, right-leaning respondents in Germany and older as well as female respondents in the UK are more willing to admit that they share fake news deliberately.

**Table 5:** Admitting of sharing of fake news by country.

	Admits deliberate sharing		Admits accidental sharing	
	(1)	(2)	(3)	(4)
	Germany	UK	Germany	UK
Deliberately shared fake news	1.585***	1.733***		
	(0.203)	(0.213)		
Accidentally shared fake news			1.802***	1.564***
			(0.137)	(0.085)
Female (ref:male)	0.707	0.594*	0.568*	0.550**
	(0.241)	(0.150)	(0.148)	(0.131)
Age	0.963*	0.943***	0.986	0.964***
	(0.014)	(0.010)	(0.011)	(0.009)
High educated (ref:low edu)	1.722+	1.338	1.493	1.312
	(0.565)	(0.326)	(0.407)	(0.294)
Separated/Single (ref:Married/Coh)	0.375*	0.669	0.622	1.077
	(0.150)	(0.183)	(0.198)	(0.263)
Middle income (ref:low)	0.301**	1.104	0.354**	1.216
	(0.134)	(0.389)	(0.131)	(0.406)
High income (ref:low)	0.199**	1.334	0.268*	1.282
	(0.115)	(0.526)	(0.138)	(0.492)
Unemployed (ref:employed)	0.945	0.512	0.274	0.596
	(1.110)	(0.595)	(0.245)	(0.624)
Out of labor force (ref:employed)	0.246*	0.955	0.492+	1.085
	(0.169)	(0.316)	(0.211)	(0.333)
Center (ref:left)	0.728	0.641	0.571+	0.571+
	(0.321)	(0.213)	(0.188)	(0.181)
Right (ref:left)	2.867*	1.862+	1.485	1.641
	(1.280)	(0.639)	(0.532)	(0.528)
N	1223	1156	1223	1156

‘Admits deliberate sharing’ is a dummy variable given value of 1 if the respondent answered ‘yes’ to the question ‘Did you ever share a political news story online that you thought at the time was made up?’ and 0 if the respondent answered ‘no’. The variable ’admits accidental sharing’ is constructed analogously, but using the question ‘Have you ever shared a political news story online that you later found out was made up?’. The variables ‘deliberately shared fake news’ and ‘accidentally shared fake news’ are scales obtained from respondents’ assessment of headlines ranging from 0 to 5 with a higher value indicating more sharing of fake news. All estimates are from logistic regression models. The coefficients represent odds ratios. HC1 robust standard errors are in parentheses. + *p* $<0.10$, * *p* $<0.05$, ** *p* $<0.01$, *** *p* $<0.001$ (two-tailed tests).

## Discussion

We constructed a novel large dataset and presented evidence on fake news detection and sharing. The large-scale surveys in Germany and the United Kingdom were administered to answer three important questions: (1) What are the individual-level determinants of the ability to detect fake news; (2) Who shares fake news deliberately and/or accidentally; and (3) Do people gauge their ability to detect fake news accurately? Our results show the importance of age, political orientation, and income in the ubiquitous problem of the spread of fake news. We document that younger and right-wing respondents share fake news more often. By contrast, the left-wing and high-income respondents are better at detecting fake news.

A deeper look into the data reveals that the majority of the fake news sharing is accidental, and a smaller fraction is deliberate. Although it is difficult to establish a causal link, the current, more stringent approach in Germany towards hate speech in social media may have made people more aware of the fake news problem and made them more cautious in sharing dubious news items, as evidenced by the differences in numbers of accidental sharing in the two countries. This policy intervention may have had a similar effect as the accuracy prompts emphasized in recent research as effective interventions^24^. An example would be participants being reminded that the accuracy of shared information is important to other participants. Our study highlights the socio-demographic groups that have the strongest tendencies for not classifying fake news correctly. At the same time, we also identify the groups that contribute through their sharing behavior to the dissemination of fake news. Overall, this study may help policymakers to efficiently target the relevant groups, e.g., through accuracy prompts.

Our results, which highlight the importance of individual characteristics in understanding recently elevated misinformation campaigns, have similarities and differences with previous findings in the literature. Guess, Nagler, and Tucker^13^ and Grinberg et al.^11^ find—similar to our analysis—that it is primarily the more conservative individuals who share fake news. Both articles argue that older cohorts share much more fake news than the younger ones, whereas the opposite is true in our  study and in Buchanan's study^14^. As in the case of Germany, Preston et al.^10^ identify higher abilities for fake news detection among those with better education. We find that those on the right on the political spectrum share fake news more often than those on the left. Similarly, Allcott and Gentzkow^4^ argued that, in the 2016 US election, fake news was widely shared in favor of Donald Trump. By contrast, Hopp, Ferrucci, and Vargo^12^ found partial support (on Facebook, but not on Twitter) to connect ideological extremity (left and right) to fake news sharing.

Previous literature has provided some potential mechanisms behind the individual-level factors. Pennycook and Rand^30^ used the dual-process model to argue that people interested in politics might contemplate more about political issues and therefore might be better in detecting fake news. We provide some suggestive evidence in the Online Appendix (Supplement Table 4). In particular, we found that respondents who are more interested in politics do indeed detect fake news better in both Germany and the UK. In addition, respondents who are more interested in politics are also more likely to share fake news in the UK, but not in Germany. Supplement Table 5 shows that this conclusion holds for both deliberate and accidental sharing. Other potential mechanisms could not be tested with our survey. For instance, Pennycook and Rand^9^ argued that it is mostly analytical thinking (or the lack of it) and not so much political orientation that matters in spreading misinformation. Similarly, Lawson and Kakkar^31^ contend that while sharing fake news is largely driven by low conscientiousness conservatives, at high levels of conscientiousness there is no difference between liberals and conservatives. Conscientiousness may also be a psychological driver behind other individual-level determinants which we have identified in our analysis, such as income or education^32^.

Finally, we found gender differences in fake news detection. In particular, it appears that females detect less fake news than men. In Supplement Table 6, we show that interest in politics cannot explain these gender differences. What appears to partly explain these gender differences is confidence in one’s ability to detect fake news. We found in Supplement Table 7 that women who are more confident in their ability to detect fake news are particularly unable to detect fake news in the UK, but not in Germany. Another possible explanation could lie in the unique role that emotional processing may play in susceptibility to fake news as found by Martel, Pennycook and Rand^33^. There is some evidence of sex differences in emotional processing^34^, although these conclusions are disputed and may be highly gendered (see for instance Shields^35^). Future research should go deeper into the psychological mechanisms behind our results and explain the sociodemographic differences in fake news detection and sharing that we have highlighted.

The usual limitations to survey research also apply to our study: respondents may misunderstand the questions, give dishonest answers, and a survey may not capture the respondents’ emotional response to the questions they encounter. Hence, we do not claim that the results are causal. Nonetheless, our results shed light on fake news detection and sharing behavior and provide timely insights to policymakers who are interested in curbing the dissemination of misinformation.

## Methods

### Data collection

Originally, we conducted large-scale surveys in four European countries: France, Germany, Spain, and the United Kingdom that we designed and programmed via Qualtrics. The surveys were administered between 3 March 2020 and 30 March 2020 by the company Respondi (https://www.respondi.com/EN/) in the national languages (French, German, Spanish and English respectively). Respondi has access to panels of representative samples of respondents to whom they email survey links. All survey questions were approved by Zayed University Research Ethics Committeee. The survey was carried out in accordance with relevant guidelines and regulations. Informed consent was obtained from all participants who were 18 years or older. We applied quotas on gender, age, income, and labor market status to create a representative survey. Once the quota had been met, the respondents could no longer submit a response. Then, in a second wave between 10 December 2021 and 14 December 2021, we contacted all respondents who had remained in the Respondi panel. The first wave of the survey dealt mostly with people’s misperceptions in the four countries, and we asked only a few questions about fake news. In the second wave, however, we included detailed questions on fake news in two countries with different political systems: Germany and the United Kingdom. Hence, in this paper, we will use the data from the second wave for these two countries.

In total, we observed 2379 individuals: 1223 in Germany and 1156 in the United Kingdom. The summary statistics alongside population values are presented in Table 1. In general, the sample appears to be representative of the population with some differences. Namely, married or cohabiting people as well as people between 18 and 35 are underrepresented in both countries. Moreover, there appear to be less people that have a high income in the United Kingdom in our sample in comparison to the population and more highly educated people in Germany. The average time to complete the survey was 21 min, and the respondents were paid only if they completed the survey. We removed respondents who did not complete the part of the questionnaire we use for the analyses and who completed the survey either very fast (in less than 5 min) or very slowly (more than two hours).

### Variables construction

We provide the complete English version of the survey in the Supplementary file. As we are interested in the determinants of fake news sharing, we first asked the respondents about individual-level characteristics. To allow for comparability with other survey-based studies, we used the standard set of individual-level variables, e.g., in the European Social Survey^36^. We constructed the following individual-level characteristics: gender (1 is female, 0 is male), age (ranges between 18 and 70), education (1 is high educated, 0 is low educated), marital status (1 is married or cohabiting, 0 is separated or single), household income (low income, middle income, high income), labor market position (employed, unemployed, out of the labor force), and political orientation (left, center, right). Education is defined as high educated if the respondents finished tertiary education and low educated if otherwise. For Germany, household income is defined as low income if the net monthly household income is below 1500 EUR, middle income between 1500 EUR and 2999 EUR, and high income if the net monthly household income is above or equal to 3000 EUR. For the UK, the corresponding thresholds refer to weekly household incomes and are: less than £400; £400–£1000; more than £1000. Political orientation is determined from the respondents’ answers to the question “In politics people sometimes talk about ‘left’ and ‘right’. Please indicate on a scale of 0–10 where you would place yourself (0 = Left; 10 = Right).”. We recategorized this variable for ease of interpretation to left (from 1 to 3), center (from 4 to 6), and right (from 7 to 10).

To measure whether respondents are able to detect fake news, we provided five fake news headlines. These headlines were taken from the DiSiNFO Database (https://euvsdisinfo.eu/disinformation-cases/) and Valencia College (https://libguides.valenciacollege.edu/c.php?g=612299 &p=4251645.). We selected headlines that avoided any national bias for the UK or Germany. None of the headlines deal with domestic affairs in Germany or the UK as this would facilitate the validation for respondents from the respective country. We also avoided headlines that could be associated with national policy stances to avoid any partisan bias. We pre-tested the headlines among five Ph.D. students to ensure some variation in the responses. The selected headlines are:

1. Pope Francis Shocks World, Endorses Donald Trump for President.
2. Israeli Defense Minister: If Pakistan sends ground troops to Syria on any pretext, we will destroy this country with a nuclear attack.
3. Macron allowed the use of Sputnik V vaccine in France.
4. Italian town forbids Christmas carols not to insult migrants.
5. Ukraine will buy the Russian vaccine from Germany at an inflated price.

We also provided five true headlines taken from Deutsche Welle^37^, The Guardian^38^, Radio France Internationale^39^, France24^40^ and CBS News^41^:

1. Donald Trump nominated for the 2021 Nobel Peace Prize.
2. Amazon had sales income of €44bn in Europe in 2020 but paid no corporation tax.
3. French homeless population doubled since 2012.
4. Switzerland ends talks with EU on co-operation agreement.
5. Iowa workers fired for refusing COVID vaccine still eligible for unemployment benefits.

The headlines were presented in text-form and not combined with any visual material, which might suggest the appearance in specific (social) media outlets. We then asked the respondents “To the best of your knowledge, how likely is it that the claim in each of the headlines is correct?” with five answer possibilities: (a) extremely unlikely, (b) somewhat unlikely, (c) neither likely nor unlikely, (d) somewhat likely, and (e) extremely likely. We operationalized fake news detection as a scale ranging from 0 to 40 with a higher value indicating better fake news detection. For fake news headlines, we gave a value of 4 if the respondent answered ‘extremely unlikely’, 3 for ‘somewhat unlikely’, 2 for ‘neither likely nor unlikely’, 1 for ‘somewhat likely’, and 0 for ‘extremely likely’. The reversed coding was used for true headlines.

To measure whether respondents share fake news, we asked “Would you consider sharing each of the following stories online (for example, through Facebook or Twitter)?”, after which we provided the same 5 fake news headlines and 5 true headlines outlined above. The respondents could choose between ‘no’, ‘maybe’, and ‘yes’. We then constructed three measures of fake news sharing. First, general fake news sharing was constructed as a scale ranging from 0 to 10 with a higher value indicating a higher propensity to share. To construct this scale, we considered the five fake news headlines and gave a value of 2 if the respondent answered ‘yes’, 1 for ‘maybe’, and 0 for ‘no’. We then aimed to distinguish between the deliberate and accidental sharing of fake news by constructing two additional scales. The first scale is deliberate sharing, meaning that the respondent recognized the fake news headlines were fake but wanted to share them anyway. Respondents recognized that a fake news headline was fake if they answered ‘extremely unlikely’ and ‘somewhat unlikely’ on the question “To the best of your knowledge, how likely is it that the claim in each of the headlines is correct?”. We consider that respondents wanted to share the fake news headlines if they answered ‘yes’ or ‘maybe’ to the question “Would you consider sharing each of the following stories online (for example, through Facebook or Twitter)?”. We then counted the number of headlines respondents were willing to share deliberately and constructed a scale ranging from 0 to 5. The second scale is accidental sharing meaning that the respondents did not recognize the fake news headlines were fake and wanted to share them. Analogous questions were used for deliberate sharing, except that not recognizing was defined as answering ‘neither likely nor unlikely’, ‘somewhat likely’, and ‘extremely likely’ on the detection of fake news questions. This scale was also constructed by counting the number of headlines respondents were willing to share accidentally, ranging from 0 to 5.

Finally, to elicit admitting of fake news sharing, we followed a public opinion poll previously conducted by Barthel, Mitchell, and Holcomb^7^. In particular, participants were asked “Have you ever shared a political news story online that you thought at the time was made up?” to measure deliberate fake news sharing and “Have you ever shared a political news story online that you later found out was made up?” to measure accidental fake news sharing. These variables are binary indicators with a value of one if the respondent answered ‘yes’ and zero if the respondent answered ‘no’. These questions were asked before the questions that included headlines.

## Methodology

We estimate how various fake news measures (fake news detection, general fake news sharing, deliberate fake news sharing, and accidental fake news sharing) relate to different individual-level characteristics using a linear model estimated by Ordinary Least Squares (OLS):

1 $$\begin{aligned} Y_{i} = \alpha + \beta \mathbf {S_{i}} + \epsilon _{i} \end{aligned}$$

In Eq. (1), $Y_{i}$ represents the fake news variables for individual *i*. The variables of interest are captured in the vector $\mathbf {S_{i}}$, representing various individual-level characteristics. These include gender (1 is female, 0 is male), age, education (1 is high educated, 0 is low educated), marital status (1 is married or cohabiting, 0 is separated or single), household income (low income, middle income, high income), labor market position (employed, unemployed, out of the labor force), and political orientation (left, center, right). We perform all analyses by country and we use HC1 robust standard errors in each specification as we have a rather large sample. The reader should keep in mind that we are not able to account for endogeneity arising from omitted factors or reverse causality and therefore do not make any causal claims. Nonetheless, we do provide timely evidence on the determinants of fake news sharing and detection.

We further analyzed the respondents’ self-perception of how they deal with fake news, namely whether the respondents admit they share fake news and how this relates to deliberate and accidental fake news sharing. For this purpose, we estimated the following model using a logistic regression:

2 $$\begin{aligned} Y_{i} = \gamma + \delta D_{i} + \tau A_{i} + \nu \mathbf {S_{i}} + \omega _{i} \end{aligned}$$

In Eq. (2), $Y_{i}$ represents an indicator given a value of 1 if the respondent themselves stated they shared fake news (deliberately or accidentally), and 0 otherwise. The parameters of interest are $\delta$ and $\tau$ representing the change in log odds of stating to have shared fake news with a one unit increase in deliberate and accidental fake news sharing respectively. However, to ease interpretation, we provide the results in odds ratios rather than log odds. If the outcome is deliberate fake news sharing, we set the parameter $\tau$ to zero, and if the outcome is accidental fake news sharing, we set the parameter $\delta$ to zero. As for the OLS models, we perform all analyses by country and we use HC1 robust standard errors in each specification.

## Supplementary Information

Supplementary Information.

## Data Availability

The data generated and analysed during the current study are available from the corresponding author on reasonable request. We are currently in the process of making the data available to the public.
