# Supplementary material for: Ability of detecting and willingness to share fake news
Source: Sci Rep. 2023 May 5;13:7298. doi: 10.1038/s41598-023-34402-6 (PMC10160725; doi:10.1038/s41598-023-34402-6)
Supplement: Supplementary file 1 — Supplementary Information. [file 41598_2023_34402_MOESM1_ESM.pdf]

Supplementary Information

Table 1. Supplement: Descriptive Statistics for Deliberate Sharers

	Shared no fake headlines	Deliberately shared at least 1 fake headline
	Mean/Prop.	Mean/Prop.
Country (ref:Germany)	.478	.546
Female	.486	.382
Age	50.7	48.6
High educated	.429	.389
Married/cohabiting	.411	.396
Low income	.217	.204
Middle income	.578	.579
High income	.204	.218
Employed	.750	.775
Unemployed	.015	.025
Out of labor force	.234	.200
Left	.202	.207
Center	.629	.550
Right	.169	.243

Table 2. Supplement: Detecting and Sharing Fake News

	(1)	(2)
	Detecting Fake News	Sharing Fake News
Country (ref:Germany)	-0.968***	0.477***
	(0.145)	(0.079)
Female (ref:male)	-0.821***	-0.213**
	(0.142)	(0.076)
Age	0.034***	-0.023***
	(0.006)	(0.004)
High educated (ref:low edu)	0.326*	-0.107
	(0.147)	(0.077)
Separated/Single (ref:Married/Coh)	0.187	-0.113
	(0.155)	(0.081)
Middle income (ref:low)	0.323+	-0.021
	(0.183)	(0.097)
High income (ref:low)	0.943***	-0.093
	(0.248)	(0.129)
Unemployed (ref:employed)	-0.385	0.060
	(0.561)	(0.367)
Out of labor force (ref:employed)	0.056	-0.148+
	(0.177)	(0.084)
Center (ref: left)	-0.742***	0.140
	(0.182)	(0.085)
Right (ref:left)	-0.758**	0.674***
	(0.234)	(0.134)
Constant	22.421***	1.841***
	(0.431)	(0.229)
N	2379	2379
adj. R-sq	0.057	0.054

Notes. All estimates are from linear models estimated by Ordinary Least Squares. HC1 robust standard errors are in parentheses. + $p<0.10$, * $p<0.05$, ** $p<0.01$, *** $p<0.001$ (two-tailed tests).

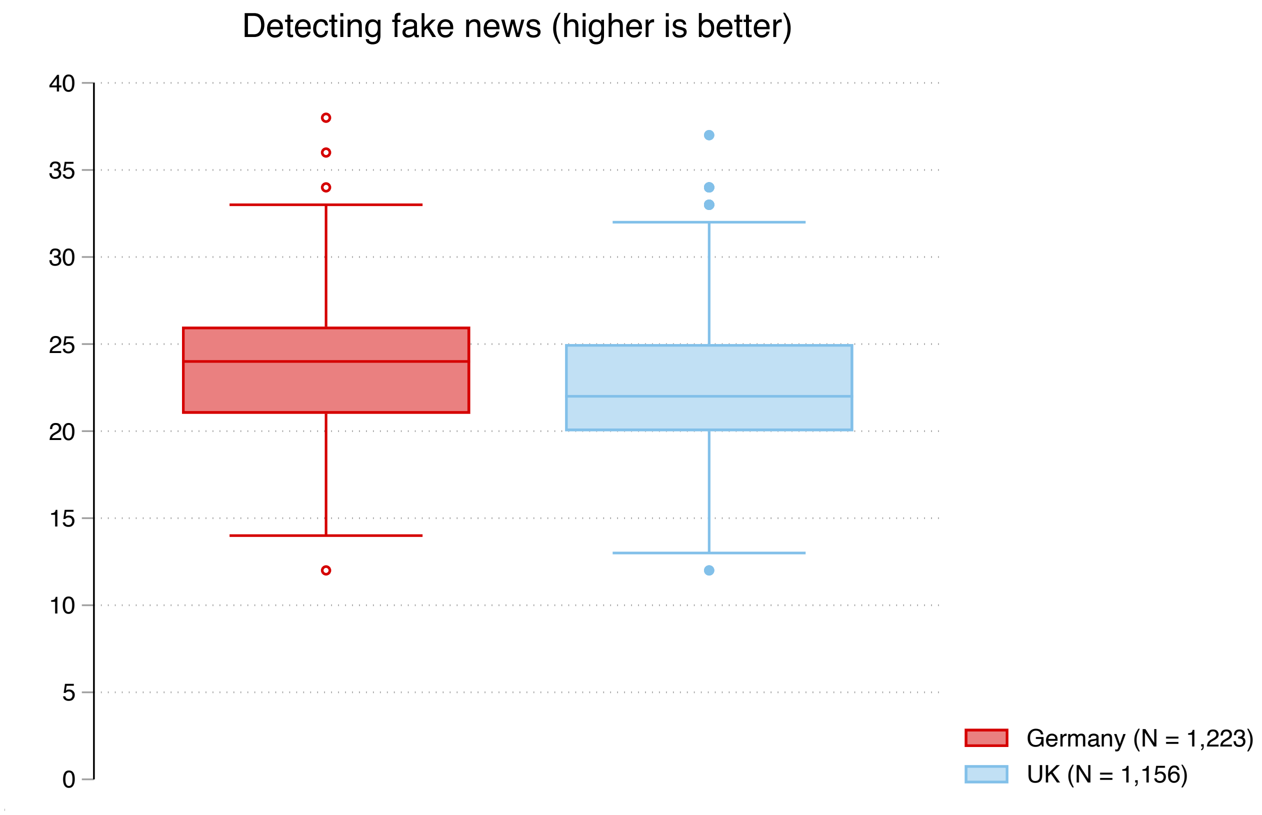

Figure 1. Supplement: Box Plot for Fake News Detection in Germany and UK

Table 3. Supplement: Sharing of True News by Country

	(1)	(2)
	Germany	UK
Female (ref:male)	-0.157	-0.327*
	(0.101)	(0.133)
Age	-0.009*	-0.042***
	(0.004)	(0.007)
High educated (ref:low edu)	-0.102	-0.061
	(0.101)	(0.135)
Separated/Single (ref:Married/Coh)	-0.082	-0.251+
	(0.112)	(0.141)
Middle income (ref:low)	-0.008	-0.089
	(0.143)	(0.158)
High income (ref:low)	-0.225	-0.257
	(0.180)	(0.209)
Unemployed (ref:employed)	0.820	-0.496
	(0.663)	(0.421)
Out of labor force (ref:employed)	-0.063	-0.060
	(0.120)	(0.148)
Center (ref:left)	-0.079	0.047
	(0.115)	(0.159)
Right (ref:left)	0.374+	0.926***
	(0.198)	(0.221)
Constant	1.509***	3.639***
	(0.286)	(0.439)
N	1223	1156
adj. R-sq	0.014	0.068

Notes. Outcome variables are scales ranging from 0 to 5 with a higher value indicating more sharing of fake news. All estimates are from linear models estimated by Ordinary Least Squares. HC1 robust standard errors are in parentheses. + p<0.10, * p<0.05, ** p<0.01, *** p<0.001 (two-tailed tests).

Table 4. Supplement: Detecting and Sharing Fake News – Political Interest

	Detecting Fake News		Sharing Fake News	
	(1)	(2)	(3)	(4)
	Germany	UK	Germany	UK
Female (ref:male)	-0.972***	-0.310	-0.065	-0.326*
	(0.200)	(0.204)	(0.094)	(0.127)
Age	0.037***	0.017+	-0.011**	-0.043***
	(0.008)	(0.009)	(0.004)	(0.006)
High educated (ref:low edu)	0.327	-0.099	-0.143	-0.111
	(0.211)	(0.207)	(0.091)	(0.127)
Separated/Single (ref:married/coh)	0.181	0.232	-0.037	-0.190
	(0.216)	(0.218)	(0.097)	(0.129)
Middle income (ref:low)	0.029	0.368	-0.000	-0.123
	(0.286)	(0.234)	(0.124)	(0.141)
High income (ref:low)	0.607	0.770*	-0.114	-0.170
	(0.387)	(0.323)	(0.160)	(0.193)
Unemployed (ref:employed)	-1.227	0.107	0.605	-0.415
	(0.784)	(0.752)	(0.637)	(0.358)
Out of labor force (ref:employed)	-0.067	0.214	-0.110	-0.106
	(0.272)	(0.235)	(0.099)	(0.132)
Center (ref: left)	-0.417+	-0.702**	-0.006	0.455**
	(0.252)	(0.261)	(0.101)	(0.145)
Right (ref:left)	-0.316	-1.072***	0.316+	1.001***
	(0.345)	(0.313)	(0.170)	(0.196)
Interest in politics	0.218***	0.156***	-0.014	0.091***
	(0.036)	(0.034)	(0.019)	(0.022)
Constant	20.900***	21.303***	1.347***	2.674***
	(0.611)	(0.608)	(0.273)	(0.426)
N	1223	1156	1223	1156
adj. R-sq	0.094	0.036	0.014	0.087

Notes. All estimates are from linear models estimated by Ordinary Least Squares. HC1 robust standard errors are in parentheses. Interest in politics is measured on a scale from 0 to 10, with a higher number indicating a higher interest in politics. The question that was posed to the survey participants was "Please indicate on a scale of 0–10 how interested you would say you are in politics (0 = Not at all interested; 10 = Very interested)." + p<0.10, * p<0.05, ** p<0.01, *** p<0.001 (two-tailed tests).

Table 5. Supplement: Deliberate and Accidental Sharing of Fake News – Political Interest

	Deliberate Sharing		Accidental Sharing	
	(1) Germany	(2) UK	(3) Germany	(4) UK
Female (ref:male)	-0.071	-0.055	-0.014	-0.183*
	(0.046)	(0.042)	(0.061)	(0.083)
Age	-0.002	-0.009***	-0.007**	-0.022***
	(0.002)	(0.002)	(0.002)	(0.004)
High educated (ref:low edu)	-0.025	-0.096*	-0.105+	-0.044
	(0.043)	(0.042)	(0.061)	(0.082)
Separated/Single (ref:Married/Coh)	0.030	-0.032	-0.067	-0.071
	(0.045)	(0.042)	(0.067)	(0.086)
Middle income (ref:low)	0.006	-0.000	-0.023	-0.110
	(0.060)	(0.048)	(0.085)	(0.099)
High income (ref:low)	0.044	0.097	-0.143	-0.219+
	(0.079)	(0.068)	(0.107)	(0.128)
Unemployed (ref:employed)	-0.001	0.159	0.238	-0.343+
	(0.129)	(0.175)	(0.310)	(0.207)
Out of labor force (ref:employed)	-0.002	0.039	-0.091	-0.114
	(0.053)	(0.054)	(0.065)	(0.092)
Center (ref:left)	-0.062	0.060	0.080	0.283**
	(0.056)	(0.048)	(0.060)	(0.098)
Right (ref:left)	-0.047	0.107+	0.235*	0.582***
	(0.066)	(0.063)	(0.101)	(0.125)
Interest in politics	-0.005	0.023***	-0.016	0.046**
	(0.010)	(0.007)	(0.013)	(0.015)
Constant	0.388**	0.569***	0.854***	1.480***
	(0.146)	(0.125)	(0.171)	(0.287)
N	1223	1156	1223	1156
adj. R-sq	-0.003	0.031	0.016	0.057

Notes. Outcome variables are scales ranging from 0 to 5 with a higher value indicating more sharing of fake news. All estimates are from linear models estimated by Ordinary Least Squares. HC1 robust standard errors are in parentheses. Interest in politics is measured on a scale from 0 to 10, with a higher number indicating a higher interest in politics. The question that was posed to the survey participants was "Please indicate on a scale of 0–10 how interested you would say you are in politics (0 = Not at all interested; 10 = Very interested)."
+ $p<0.10$, * $p<0.05$, ** $p<0.01$, *** $p<0.001$ (two-tailed tests).

Table 6. Supplement: Detecting and Sharing Fake News – Political Interest and Gender Differences

	Detecting Fake News		Sharing Fake News	
	(1) Germany	(2) UK	(3) Germany	(4) UK
Female (ref:male)	-0.333	0.285	-0.474+	-0.542+
	(0.493)	(0.416)	(0.256)	(0.278)
Age	0.037***	0.017+	-0.011**	-0.043***
	(0.008)	(0.009)	(0.004)	(0.006)
High educated (ref:low edu)	0.324	-0.112	-0.141	-0.106
	(0.211)	(0.207)	(0.091)	(0.127)
Separated/Single (ref:married/coh)	0.184	0.247	-0.040	-0.196
	(0.216)	(0.218)	(0.097)	(0.129)
Middle income (ref:low)	0.036	0.359	-0.005	-0.120
	(0.287)	(0.234)	(0.125)	(0.141)
High income (ref:low)	0.620	0.763*	-0.123	-0.168
	(0.387)	(0.323)	(0.159)	(0.193)
Unemployed (ref:employed)	-1.184	0.060	0.577	-0.398
	(0.770)	(0.753)	(0.637)	(0.360)
Out of labor force (ref:employed)	-0.056	0.207	-0.117	-0.103
	(0.272)	(0.235)	(0.100)	(0.132)
Center (ref: left)	-0.410	-0.692**	-0.010	0.451**
	(0.252)	(0.260)	(0.102)	(0.146)
Right (ref:left)	-0.300	-1.080***	0.307+	1.004***
	(0.345)	(0.312)	(0.169)	(0.196)
Interest in politics	0.271***	0.204***	-0.048+	0.074*
	(0.058)	(0.046)	(0.027)	(0.031)
Female x Interest in politics	-0.095	-0.097	0.061+	0.035
	(0.070)	(0.065)	(0.034)	(0.043)
Constant	20.490***	20.979***	1.609***	2.791***
	(0.689)	(0.626)	(0.303)	(0.454)
N	1223	1156	1223	1156
adj. R-sq	0.095	0.037	0.016	0.086

Notes. All estimates are from linear models estimated by Ordinary Least Squares. HC1 robust standard errors are in parentheses. Interest in politics is measured on a scale from 0 to 10, with a higher number indicating a higher interest in politics. The question that was posed to the survey participants was "Please indicate on a scale of 0–10 how interested you would say you are in politics (0 = Not at all interested; 10 = Very interested)." + p<0.10, * p<0.05, ** p<0.01, *** p<0.001 (two-tailed tests).

Table 7. Supplement: Detecting and Sharing Fake News – Confidence and Gender Differences

	Detecting Fake News		Sharing Fake News	
	(1) Germany	(2) UK	(3) Germany	(4) UK
Female (ref:male)	-1.132+	0.856	-0.582+	-0.225
	(0.612)	(0.654)	(0.297)	(0.432)
Age	0.046***	0.022*	-0.011**	-0.039***
	(0.008)	(0.009)	(0.004)	(0.006)
High educated (ref:low edu)	0.442*	-0.008	-0.170+	-0.061
	(0.211)	(0.205)	(0.088)	(0.127)
Separated/Single (ref:Married/Coh)	0.152	0.202	-0.047	-0.186
	(0.219)	(0.220)	(0.097)	(0.131)
Middle income (ref:low)	0.060	0.479*	-0.030	-0.092
	(0.288)	(0.234)	(0.126)	(0.142)
High income (ref:low)	0.708+	0.893**	-0.164	-0.133
	(0.390)	(0.327)	(0.159)	(0.194)
Unemployed (ref:employed)	-1.098	-0.032	0.555	-0.496
	(0.837)	(0.734)	(0.628)	(0.383)
Out of labor force (ref:employed)	0.009	0.198	-0.117	-0.083
	(0.272)	(0.237)	(0.100)	(0.131)
Center (ref: left)	-0.499*	-0.891***	0.013	0.379**
	(0.251)	(0.261)	(0.102)	(0.145)
Right (ref:left)	-0.343	-1.074***	0.307+	1.002***
	(0.349)	(0.313)	(0.168)	(0.196)
Confidence	0.213*	0.149*	-0.038	0.115*
	(0.085)	(0.073)	(0.038)	(0.047)
Female x Confidence	0.017	-0.204*	0.091+	-0.019
	(0.102)	(0.101)	(0.048)	(0.070)
Constant	20.640***	21.117***	1.527***	2.328***
	(0.745)	(0.756)	(0.369)	(0.483)
N	1223	1156	1223	1156
adj. R-sq	0.084	0.024	0.017	0.081

Notes. All estimates are from linear models estimated by Ordinary Least Squares. HC1 robust standard errors are in parentheses. Confidence is measured on a scale from 0 to 10, with a higher number indicating a higher confidence in one's ability to detect fake news. The question that was posed to the survey participants was "Please indicate on a scale of 0–10 how confident you are in your own ability to recognize news that is made up (0 = Not at all confident; 10 = Very confident)." + p<0.10, * p<0.05, ** p<0.01, *** p<0.001 (two-tailed tests).

Supplement: UK version of the questionnaire

Q1 Were you born in the United Kingdom? Yes / No

Q2 What is your gender? Male / Female / Non-Binary

Q3 What is your age?

Q4 How long have you lived in your current city? (years)

Q5 What is your gross weekly household income? Less than £400 / £400–£600 / £600–£1.000 / More than £1.000

Q6 Please indicate your marital status. Single / Couple, Married / Separated or Divorced / Widowed

Q7 How many children do you have? I do not have children / 1 / 2 / 3 / 4 / 5 / More than 5

Q8 Which category best describes your highest level of education? Compulsory Education / High School / University (but not finished) / Bachelor Degree / Master Degree / Doctoral Degree

Q9 In the last survey, you estimated population share of immigrants as .... In 2019, population share of immigrants in the UK was 14%.

Q10 In the last survey, you estimated income share of the richest 10% as .... In 2019, income share of the richest 10% in the UK was 26%.

Q11 Which of these descriptions best describes your situation? Please select ONLY one. In paid work / In education / Self employed / Unemployed and actively looking for a job / Unemployed, wanting a job but not actively looking for a job / Permanently sick or disabled / Retired / In community or military service / Doing housework, looking after children or other persons / Refusal/Don't know

Q12 Have you ever had a paid job? Yes / No / Refusal/Don't know

Q13 In what year were you last in a paid job? Write in year / Refusal/Don't know

Q14 In your main job are/were you. . . Please select ONLY one. An employee / Self-employed / Working for your own family's business / Refusal/Don't know

Q15 How many employees (if any) do/did you have? Write in the number of employees / Refusal/Don't know

Q16 Do/did you have a work contract of...Unlimited duration / Limited duration / Do/did you have no contract? / Refusal/Don't know

Q17 Including yourself, about how many people are/were employed at the place where you usually work/worked...Under 10 / 10 to 24 / 25 to 99 / 100 to 499 / 500 or more / Refusal/Don't know

Q18 Have you ever been unemployed and seeking work for a period of more than three months in the last five years? Yes / No / Refusal/Don't know

Q19 Have any of these periods lasted for 6 months or more? Yes / No / Refusal/Don't know

Q20 Please consider the total income of all household members. What is the main source of income in your household? Wages or salaries / Income from self-employment / Pensions / Unemployment/redundancy benefit / Any other social benefits or grants / Income from investment, savings, insurance or property / Income from other sources / Refusal/Don't know

Q21 Which of the descriptions comes closest to how you feel about your household's income nowadays? Living comfortably on present income / Coping on present income / Finding it difficult on present income / Finding it very difficult on present income / Refusal/Don't know

Q22 Please indicate on a scale of 0–10 how interested you would say you are in politics (0 = Not at all interested; 10 = Very interested).

Q23 Please indicate on a scale of 0–10 how much you would say the political system in the United Kingdom allows people like you to have a say in what the government does (0 = Not at all; 10 = A great deal).

Q24 Please indicate on a scale of 0–10 how much you personally trust each of these institutions (0 = Do not trust at all; 10 = Complete trust). Country's parliament / The legal system / The police / Politicians / Political parties / The European Parliament / The United Nations

Q25 Some people don't vote nowadays for one reason or another. Did you vote in the last national election in December 12th, 2019? Yes / No / Refusal/Don't know

Q26 Which party did you vote for in that election? Conservative / Labour / Liberal Democrat / UKIP / Paid Cymru / Green Party / SNP / Brexit Party / Other (write in) / Refusal/Don't know

Q27 Which party do you plan to vote in the next national election? Conservative / Labour / Liberal Democrat / UKIP / Paid Cymru / Green Party / SNP / Brexit Party / Other (write in) / Refusal/Don't know

Q28 In politics people sometimes talk about "left" and "right". Please indicate on a scale of 0–10 where you would place yourself (0 = Left; 10 = Right).

Q29 Please indicate on a scale of 0–10 how religious you think you are (0 = Not religious at all; 10 = Very religious).

Please indicate on a scale of 0–10 whether you agree or disagree with the following statements (0 = Completely disagree; 10 = Completely agree).

Q30 The opinion of ordinary people is worth more than that of experts and politicians.

Q31 Politicians should listen more closely to the problems the people have.

Q32 Ministers should spend less time behind their desks, and more among the ordinary people.

Q33 People who have studied for a long time and have many diplomas do not really know what makes the world go round.

For the next two questions, notice that we consider an ethnic group as a community or population made up of people who share a common cultural background.

Q34 Please indicate on a scale of 0–10 to what extent you think the United Kingdom should allow people of the same race or ethnic group than the majority of the British people to come and live here (0 = Allow none; 10 = Allow many to come and live here).

Q35 Please indicate on a scale of 0–10 to what extent you think the United Kingdom should allow people of the different race or ethnic group than the majority of the British people to come and live here (0 = Allow none; 10 = Allow many to come and live here).

Q36 Please indicate on a scale of 0–10 to what extent you think the United Kingdom should allow people of different religious faith than the majority of the British people to come and live here (0 = Allow none; 10 = Allow many to come and live here).

Q37 Please indicate on a scale of 0–10 to what extent you think the United Kingdom should allow people from poorer countries outside Europe to come and live here (0 = Allow none; 10 = Allow many to come and live here).

Q38 Please indicate on a scale of 0–10 to what extent you think the United Kingdom has become a worse or a better place to live by people coming to live here from other countries (0 = Worse place to live; 10 = Better place to live).

Q39 Typically, how often do you access news? By news we mean national, international, regional/local news and other topical events accessed via radio, TV, newspaper or online. Several times a day / Once a day / Several times a week / Once a week / Several times a month / Once a month / Less often than once a month / Whenever I come across by coincidence / Almost never / Never

Thinking about your news habits, please indicate on a scale of 0-10 how often do you. . . (0 = Never; 10 = Always).

Q40 Read any newspapers in print?
Q41 Listen to news on the radio?
Q42 Watch television news?
Q43 Get news from a social media site (such as Facebook, Twitter, or Snapchat)?
Q44 Get news from a news website or app?
Q45 Which, if any, of the following sources of information do you use to keep up with political issues? Please select all that apply. Friends, relatives or colleagues / National printed newspapers and/or their online sites/apps / Radio broadcasters and/or online sites/apps / TV broadcasters and/or online sites/apps / Politically focused magazines and/or online sites/apps / Political parties and/or their newsletters or online sites / Online specialist sites or political blogs / Social media such as Facebook and Twitter / Don't know / None of these
Q46 Please indicate on a scale of 0-10 how much trust and confidence you have in the mass media – such as newspapers, TV and radio – when it comes to reporting the news fully, accurately and fairly (0 = None at all; 10 = A great deal).
Q47 Please indicate on a scale of 0–10 how much trust and confidence you have in the social media – such as Twitter, Facebook, Instagram and YouTube – when it comes to reporting the news fully, accurately and fairly (0 = None at all; 10 = A great deal).
Q48 On a typical day, about how much time do you spend using the internet on a computer, tablet, smartphone or other device, whether for work or personal use? Please give your answer in hours and minutes.
Q49 Please indicate on a scale of 0–10 how often you come across news stories about politics online that you think are not fully accurate (0 = Never; 10 = Always).
Q50 Have you ever shared a political news story online that you later found out was made up? Yes / No / No answer
Q51 Have you ever shared a political news story online that you thought at the time was made up? Yes / No / No answer
Q52 To the best of your knowledge, how likely is it that the claim in each of the below headlines is correct? (Extremely unlikely / Somewhat unlikely / Neither likely nor unlikely / Somewhat likely / Extremely likely)
Pope Francis Shocks World, Endorses Donald Trump for President / Israeli Defense Minister: If Pakistan send group troops to Syria on any pretext, we will destroy this country with a nuclear attack / Macron allowed the use of Sputnik V vaccine in France / Italian town forbids Christmas carols not to insult migrants / Ukraine will buy the Russian vaccine from Germany at an inflated price / Donald Trump nominated for the 2021 Nobel Peace Prize / Amazon had sales income of €44bn in Europe in 2020 but paid no corporation tax / French homeless population doubled since 2012 / Switzerland ends talks with EU on co-operation agreement / Iowa workers fired for refusing COVID vaccine still eligible for unemployment benefits
Q53 Would you consider sharing each of the following stories online (for example through Facebook or Twitter)? (No, Maybe, Yes)
Pope Francis Shocks World, Endorses Donald Trump for President / Israeli Defense Minister: If Pakistan send group troops to Syria on any pretext, we will destroy this country with a nuclear attack / Macron allowed the use of Sputnik V vaccine in France / Italian town forbids Christmas carols not to insult migrants / Ukraine will buy the Russian vaccine from Germany at an inflated price / Donald Trump nominated for the 2021 Nobel Peace Prize / Amazon had sales income of €44bn in Europe in 2020 but paid no corporation tax / French homeless population doubled since 2012 / Switzerland ends talks with EU on co-operation agreement / Iowa workers fired for refusing COVID vaccine still eligible for unemployment benefits
Q54 Please indicate on a scale of 0–10 how confident you are in your own ability to recognize news that is made up (0 = Not at all confident; 10 = Very confident).
Q55 Please indicate on a scale of 0–10 how much you think these kinds of news stories leave people confused about the basic facts of current issues and events (0 = Not at all; 10 = A great deal).
Please indicate on a scale of 0–10 whether you agree or disagree with the following statements (0 = Completely disagree; 10 = Completely agree).
Q56 There is too much moral decay today.
Q57 The sense of belonging together that we used to have is irrevocably lost.
Q58 Parents no longer adequately educate their children.
Q59 People don't care for each other any more.
Q60 The United Kingdom will face a situation of ever-increasing job insecurity.
Q61 Even more enterprises will move to low-wage countries, threatening employment in the United Kingdom.
Q62 In order to face the competition of other countries we will have to dismantle our welfare state.
Q63 Multinational enterprises will become increasingly powerful, small enterprises are bound to suffer.
Q64 Opening the European frontiers means that our employers will prefer the low-cost workers from poorer countries to our own workers.
Q65 In the future we will become even less open and tolerant with regard to people from other cultures.
Q66 The relationship between Christians and Muslims is bound to become violent in the future.
Q67 The relationship between Christians and Jews is bound to become violent in the future.
Q68 Please indicate on a scale of 0–10 whether you agree or disagree with the following statement (0 = Completely disagree; 10 = completely agree). You can generally trust the people who run our government to do what is right.
Q69 For the next question, please consider globalization as the increased trade between countries in goods, services, and investments. Please indicate on a scale of 0–10 whether you think globalization has had a negative or a positive effect on each of the following (0 = Completely negative effect; 10 = Completely positive effect). British factory workers / Multinational corporations based in the United Kingdom / You and your immediate family / The British economy
Please indicate on a scale of 0–10 whether you agree or disagree with the following statements (0 = Completely disagree; 10 = Completely agree).
Q70 It is important to live in secure and safe surroundings.
Q71 People should follow rules at all times, even when no-one is watching.
Q72 It is important that the government is strong and ensures safety against all threats.
Q73 It is important to follow traditions and customs handed down by religion or family.
In the following four questions, we refer to legal immigrants as people who were not born in the United Kingdom and legally moved here at a certain point of their life. We are NOT considering irregular migration.
Q74 Think about all of the currently living in the United Kingdom. Out of every 100 people in the United Kingdom, how many are born in another country?
Q75 Fill in the boxes below to indicate how many out of every 100 people in the United Kingdom you think practice each religion. Christianity / Islam / Buddhism / Hinduism / Other Religions/Atheist/No religious affiliation

Q76 Out of every 100 people, who are between 20 and 64 years old, in the United Kingdom how many are currently unemployed? By unemployed we mean people who are currently not working but searching for a job (and maybe unable to find one). Now let's compare this to the number of unemployed among foreign-born people. Out of every 100 foreign-born people how many do you think are currently unemployed?

Q77 The poverty line is the estimated minimum level of income needed to secure the necessities of life. Out of every 100 adult people born in the United Kingdom, how many live below the poverty line? Let's compare this to poverty among legal immigrants. Out of every 100 legal immigrants in the United Kingdom today, how many do you think live below the poverty line?

Q78 The International Organization for Migration (IOM) defines irregular migration as "movement that takes place outside the regulatory norms of the sending, transit and receiving country". A migrant in an irregular situation may fall within one or more of the following circumstances: He or she may enter the country irregularly; he or she may reside in the country irregularly; he or she may be employed in the country irregularly. Think about the evolution of the irregular migration flows in Europe in the last 3 years. It has increased over time / It has decreased over time / It has kept constant over time / Don´t know

Q79 Think about the evolution of detections of illegal border crossing at the EU's external borders in the last 3 years. It has increased over time / It has decreased over time / It has kept constant over time / Don´t know

Q80 How many Islamist terrorists do you think have been arrested in the United Kingdom in 2020?

Q81 How many people do you think have been killed during terror attacks committed by Islamist terrorists in the United Kingdom in the last 5 years?

Q82 Please indicate on a scale of 0–10 whether you agree or disagree with the following statement (0 = Completely disagree; 10 = Completely agree). Most crimes in the UK are committed by foreigners.

Q83 What percentage of the prison population in the United Kingdom are foreign national prisoners?

Q84 What do you think is the income share of the poorest 20% of all people living in the United Kingdom?

Q85 What do you think is the income share of the richest 10% of all people living in the United Kingdom?

Q86 How large is the share of taxes and social contributions in percentage of GDP (Gross Domestic Product) in the United Kingdom?

Q87 According to the share of taxes and social contributions as a percentage of GDP, in which position do you think the United Kingdom is among the 27 European Union countries and the UK (28 countries in total)? Notice that a higher position in the list implies a larger share.

For the next questions, please consider corruption in a broad sense, including offering, giving, requesting and accepting bribes or kickbacks, valuable gifts and important favors, as well as any abuse of power for private gain.

Q88 Transparency International is the leading global civil organization on the fight against corruption. Each year they elaborate a Corruption Perceptions Index which ranks 180 countries and territories by their perceived levels of public sector corruption according to experts and business people. In which position do you think the United Kingdom is among the 27 European Union countries and the UK (28 countries in total)?

Q89 There are people who tend to be towards the top of our society and people who tend to be towards the bottom. On a scale of 0–10 Where you would put yourself (0 = Bottom of our society; 10 = Top of our society).

Please indicate on a scale of 0–10 to what extent you agree with the following statements (0 = Completely disagree; 10 = Completely agree).

Q90 I experience a general sense of emptiness.

Q91 There are many people I can trust completely.

Q92 I miss having people around me.

Q93 I often feel rejected.

Q94 I have enough opportunities to advance in life.

Q95 I know exactly where I feel at home and where I belong.
